# Readability and Quality of Online Patient Education Materials Concerning Posterior Cruciate Ligament Reconstruction

**DOI:** 10.7759/cureus.58618

**Published:** 2024-04-19

**Authors:** Michele Venosa, Simone Cerciello, Mohammad Zoubi, Giuseppe Petralia, Andrea Vespasiani, Massimo Angelozzi, Emilio Romanini, Giandomenico Logroscino

**Affiliations:** 1 Department of Life, Health and Environmental Sciences, University of L'Aquila, L'Aquila, ITA; 2 Department of Orthopaedics, RomaPro, Polo Sanitario San Feliciano, Rome, ITA; 3 Department of Orthopaedics and Traumatology, Fondazione Policlinico Universitario Agostino Gemelli, Istituto di Ricovero e Cura a Carattere Scientifico (IRCCS), Rome, ITA; 4 Orthopaedic Department, Casa di Cura Villa Betania, Rome, ITA; 5 Department of Orthopaedics, Italian Working Group on Evidence-Based Orthopaedics (GLOBE), Rome, ITA; 6 Department of Minimally Invasive and Computer-Assisted Orthopaedic Surgery, San Salvatore Hospital, L'Aquila, ITA

**Keywords:** patient education, internet, information quality, readability, posterior cruciate ligament (pcl) reconstruction

## Abstract

Objective

This study aimed to assess the quality of online patient educational materials regarding posterior cruciate ligament (PCL) reconstruction.

Methods

We performed a search of the top-50 results on Google® (terms: “posterior cruciate ligament reconstruction,” “PCL reconstruction,” “posterior cruciate ligament surgery,” and “PCL surgery”) and subsequently filtered to rule out duplicated/inaccessible websites or those containing only videos (67 websites included). Readability was assessed using six formulas: Flesch-Kincaid Reading Ease (FRE), Flesch-Kincaid Grade Level (FKG), Gunning Fog Score (GF), Simple Measure of Gobbledygook (SMOG) Index, Coleman-Liau Index (CLI), Automated Readability Index (ARI); quality was assessed using the JAMA benchmark criteria and recording the presence of the HONcode seal.

Results

The mean FRE was 49.3 (SD 11.2) and the mean FKG level was 8.09. These results were confirmed by the other readability formulae (average: GF 8.9; SMOG Index 7.3; CLI 14.7; ARI 6.5). A HONcode seal was available for 7.4 % of websites. The average JAMA score was 1.3.

Conclusion

The reading level of online patient materials concerning PCL reconstruction is too high for the average reader, requiring high comprehension skills.

Practice implications

Online medical information has been shown to influence patient healthcare decision processes. Patient-oriented educational materials should be clear and easy to understand.

## Introduction

Internet resources play a key role in the spread of health information, so patient involvement is a leading marker for decision-making outcomes [1,2]. The active role played by patients is mediated by the quality and readability of Internet resources, and disentangling this network is not simple [3]. Quality healthcare can sprout from an uplifting discussion between health professionals and patients. This collaborative knowledge can improve health professionals' approach to mutual interaction for a shared decision-making process [4]. In a patient-centered delivery model, accessing the right health information online can help patients become active participants in the information process [2]. For this reason, improving health literacy among patients, by translating academic knowledge and information into a more practical pattern, is vital to support this conscious interaction. According to a report issued by Eligibility.com in 2019, 89% of Americans “googled” their health information before going to an actual doctor [5]. Online health searches have been increasing during the COVID-19 pandemic, and a growing trend is expected in the next years as well. According to Google LLC management, about 7% of Google®’s daily searches are health-related with over 70,000 search queries every minute and over one billion every day.

Different studies have examined the readability and quality of online educational material in different orthopedic subspecialties, including specific knee pathologies and their surgical treatment [6-16]. Anyway, up to date, no study has investigated the quality and readability of online information concerning posterior cruciate ligament (PCL) reconstruction. PCL injuries can be a consequence of sports activities or traffic accidents and have a major impact not only on individual lives but also on society, considering the costs of care and the prolonged loss of productivity [17,18]. PCL reconstruction by tendon grafting can restore physiologic knee function, allowing a return to common daily activities and sports practice and delaying the onset and evolution of knee osteoarthritis.

Even though this argument has a certain interest in the general population, as confirmed by the myriad of results of a Google® search query on this argument, we did not trust the good quality of online resources concerning PCL reconstruction, similar to other investigated orthopedic topics [19]. The goal of this study was to confirm or disprove our preliminary impression.

## Materials and methods

Search strategy

Preliminary research to determine the total market share of the most popular search engines on the World Wide Web was carried out. The top search engine was Google® (Google Inc., Mountain View, CA), accounting for 91.43% of the market share worldwide (https://gs.statcounter.com/search-engine-market-share). Considering its distinct advantage over the other search engines this platform was the only one selected for the purpose of the present study. The search was performed by two independent researchers on ­June 30, 2023, at 15:30 (geographic area set on Google®: United States). Google Chrome was selected as the browser, and the anonymous modality in the search engine was used to prevent the potential bias determined by cookies, caches, and temporary Internet files. The terms used in the search string were “posterior cruciate ligament reconstruction,” “PCL reconstruction,” “posterior cruciate ligament surgery,” and “PCL surgery,” and the top 50 results per search term were collected. This collection was subsequently filtered so that all peer-reviewed journal-related web pages, all duplicated or inaccessible websites, or those containing only videos or advertisements for commercial purposes were removed. At the end of this process, 67 unambiguous websites providing educational materials to patients, instead of healthcare providers or medical professionals, were chosen (Table 1).

**Table 1:** List of the top websites on Google. The terms used in the search string are “posterior cruciate ligament reconstruction,” “PCL reconstruction,” “posterior cruciate ligament surgery,” and “PCL surgery."

#	Website link
1	https://drrobertlaprademd.com/pcl-reconstruction/
2	https://www.orthovirginia.com/posterior-cruciate-ligament-pcl-reconstruction/
3	https://www.ypo.education/orthopaedics/knee/posterior-cruciate-ligament-reconstruction-t59/video/
4	https://www.physio-pedia.com/PCL_Reconstruction
5	https://www.wheelessonline.com/joints/posterior-cruciate-ligament-reconstruction/
6	https://www.mercy.com/health-care-services/orthopedics-sports-medicine-spine/specialties/knee-leg/treatments/pcl-surgery
7	https://www.orthopaedicsurgery.uci.edu/pcl-reconstruction-orthopaedic-irvine-newportbeach-california.html
8	https://manhattansportsdoc.com/pcl-reconstruction-posterior-cruciate-ligament-orthopedic-knee-surgeon-manhattan-new-york-city-ny/
9	https://www.faoconline.com/home/procedures/pcl-reconstruction
10	https://www.sportssurgerychicago.com/knee/pcl-reconstruction-surgery-westchester-oakbrook-hinsdale-il/
11	https://noyeskneeinstitute.com/specialties-and-programs/pcl-reconstruction/
12	https://rileywilliamsmd.com/posterior-cruciate-ligament-pcl-reconstruction-repair-manhattan-new-york-city-ny/
13	https://www.brianforsythemd.com/pcl-reconstruction%E2%80%93allograft.html
14	https://www.tigerortho.com/pcl-reconstruction-orthopaedic-shoulder-knee-sports-medicine-surgeon-boston-ma.html
15	https://www.atlanticortho.com/pcl-reconstruction/
16	https://www.iskinstitute.com/kc/knee/pcl_tear/t3.html
17	https://srosm.com/orthopedic-specialties/knee/knee-treatments/pcl-reconstruction/
18	https://www.joecoopermd.com/contents/additional-services/knee/pcl-tear-reconstruction
19	https://www.orthozane.com/Specialties/Knee/Arthroscopic-PCL-Reconstruction/
20	https://www.wkhs.com/health-resources/wk-health-library/medical-procedures-tests-care-and-management/a-z/posterior-cruciate-ligament-(pcl)-reconstruction
21	https://lasportsorthomd.com/pcl-reconstruction-surgery-van-nuys-thousand-oaks-los-angeles-ca/
22	https://www.orthobullets.com/knee-and-sports/3009/pcl-injury
23	https://www.alfredmansourmd.com/posterior-cruciate-ligament-reconstruction-alfred-a-mansour-md-hip-preservation-sports-medicine-surgeon.html
24	https://www.drlingarajkrishna.com/pcl-reconstruction
25	https://matthewprovenchermd.com/pcl-reconstruction-surgery-vail-aspen-denver-co/
26	https://www.timothybertmd.com/pcl-reconstruction-sports-medicine-scottsdale-phoenix-mesa.html
27	https://rothmanortho.com/specialties/treatments/posterior-cruciate-ligament-pcl-reconstruction
28	https://orthosportandspine.com/treatments/pcl-reconstruction/
29	https://www.advancedosm.com/pcl-reconstruction-orthopaedic-sports-medicine-specialist-cypress-houston-tx/
30	https://www.physio-pedia.com/Posterior_Cruciate_Ligament_Injury
31	https://www.sports-health.com/sports-injuries/knee-injuries/posterior-cruciate-ligament-pcl-tear-treatment-options
32	https://www.scottfaucettmd.com/conditions-and-treatments/knee/posterior-cruciate-ligament-reconstruction-washington-dc/
33	https://my.clevelandclinic.org/health/diseases/21793-pcl-posterior-cruciate-ligament-tears
34	https://www.uptodate.com/contents/posterior-cruciate-ligament-injury
35	https://www.choosept.com/guide/physical-therapy-guide-posterior-cruciate-ligament-injury
36	https://www.fxrxinc.com/contents/additional-services/knee-treatment/posterior-cruciate-ligament-reconstruction
37	https://www.bonsecours.com/health-care-services/orthopedics-sports-medicine/knee/treatments/pcl-surgery
38	https://www.winchesterhospital.org/health-library/article?id=916896
39	https://www.missoulaboneandjoint.com/arthroscopic-posterior-cruciate-ligament-repair-surgery/
40	https://www.stgeorgesurgical.com/procedure/posterior-cruciate-ligament-pcl-repair/
41	https://medlineplus.gov/ency/patientinstructions/000680.htm
42	https://www.shorelineortho.com/specialties/knee-pcl_posterior_cruciate_ligament_injuries_and_reconstruction.php
43	https://www.orthopedicsurgeonnyc.com/pcl-reconstruction-surgery
44	https://emedicine.medscape.com/article/1252128-overview
45	https://centralcoastortho.com/patient-education/posterior-cruciate-ligament-pcl-reconstruction/
46	https://www.eliteorthopaedic.com/contents/patient-education/posterior-cruciate-ligament-pcl-tear
47	https://www.jorgechahlamd.com/knee/pcl-tear-chicago-il/
48	https://watsonorthopaedics.com/home/health-professional/knee/posterior-cruciate-injury-and-recon/
49	https://www.lahey.org/health-library/posterior-cruciate-ligament-pcl-repair/
50	https://www.shouldersandknees.com/posterior-cruciate-ligament-pcl-reconstruction/
51	https://www.sportssurgerynewyork.com/posterior-cruciate-ligament-pcl-reconstruction-sports-medicine-specialist-ny.html
52	https://www.sportsmd.com/sports-injuries/knee-injuries/pcl-tear/
53	https://www.lmh.org/get-care/orthokansas/knee-leg/pcl-tear-reconstruction/
54	https://www.webmd.com/fitness-exercise/posterior-cruciate-ligament-injury
55	https://www.proortho.com/knee/pcl-surgery/
56	https://www.marshfieldclinic.org/specialties/orthopedics/knee-surgery/pcl
57	https://www.cincysportssurgeon.com/pcl-tears-sports-medicine-orthopedic-surgeon.html
58	https://robertboykinmd.com/pcl-reconstruction-asheville-north-carolina/
59	https://www.joionline.net/trending/content/what-recovery-time-pcl-tear
60	https://www.uofmhealth.org/conditions-treatments/cmc/knee/pcl
61	https://www.bethesdaccsc.com/pcl-injuries-surgery-center-maryland.html
62	https://treasurevalleyhospital.com/PCL-Surgery
63	https://www.ortho-sa.com/pcl-reconstruction-orthopedic-surgeons-san-antonio-tx.html
64	https://www.angelinaveramd.com/pcl-reconstruction-orthopedic-sports-medicine-specialist-las-vegas-summerlin-henderson.html
65	https://www.rachelfrankmd.com/pcl-injuries-orthopaedic-surgeon-sports-medicine-specialist-denver-co.html
66	https://www.howardluksmd.com/pcl-injury-and-surgery-reconstruction/
67	https://www.katyorthopaedics.com/pcl-reconstruction-dr-volkan-surgeon-fort-bend-cypress-tx.html

The readability (indicating the level of education required to read text with ease) of each website was performed by using a specific platform available on the Internet [20]. This platform can analyze and extract data from websites to produce different reading scores: Flesch-Kincaid Reading Ease (FRE), Flesch-Kincaid Grade Level (FKG), Gunning Fog Score (GF), Simple Measure of Gobbledygook (SMOG) Index, Coleman-Liau Index (CLI), and Automated Readability Index (ARI).

These scales (developed for the United States educational context) are based on different formulas to determine a readability grade (Table 2).

**Table 2:** Readability scores' formulas Source: [20]

Readability score	Readability formulas
Flesch-Kincaid Reading Ease	206.835 - 1.015 x (words/sentences) - 84.6 x (syllables/words)
Flesch-Kincaid Grade Level	0.39 x (words/sentences) + 11.8 x (syllables/words) - 15.59
Gunning Fog Score	0.4 x ( (words/sentences) + 100 x (complexWords/words) )
Simple Measure of Gobbledygook (SMOG) Index	1.0430 x sqrt( 30 x complexWords/sentences ) + 3.1291
Coleman-Liau Index	5.89 x (characters/words) - 0.3 x (sentences/words) - 15.8
Automated Readability Index	4.71 x (characters/words) + 0.5 x (words/sentences) - 21.43

Readability scores and quality tests

The Flesch-Kincaid readability scores (reading ease and grade level) are the most widely used tools for readability. The FRE assigns a score ranging from 1 to 100, where 100 signifies the highest readability score (a very easy-to-read text, easily understood by an average 11-year-old student) to a text. A score between 70 and 80 corresponds to school grade level 8. The FKG expresses the approximate reading grade level of a text: when a text has an FKG score of 8, the reader needs a grade level of 8 to read and understand its meaning.

The GF score estimates the years of formal education needed to comprehend a text on the first reading, with a score ranging between 0 and 20. A text aimed at the average reader should match with a grade level of around 8.

The SMOG index was created by a clinical psychologist (G Harry McLaughlin) and designed to estimate the years of education a person needs to comprehend writing. It is widely used, especially in the healthcare sector, and it is best to assess texts of 30 sentences or more. A score under 9 is suggestive of an easy-to-read text (education level required: middle school).

The CLI evaluates the United States grade level necessary to understand a text. A text aimed at a generalist audience should correspond to a grade level of around 8-10.

The ARI measures how easy it is to understand a text and gives an estimate of the US grade level necessary to comprehend a passage, with a score ranging from 1 (very easy text) to 14 (complex text). A score under 8 should be desirable to grant a wide comprehension of the text in the general population.

In addition, the quality of the information provided by the selected websites was evaluated, using two validated tools: the Journal of the American Medical Association (JAMA) benchmark and the Health on the Net Foundation (HON) code certification.

The JAMA benchmark criteria, proposed by Silberg et al. in 1997 [21], are simple tools to verify how much the source of information is credible and reasonable. They are based on four parameters: authorship, attribution, disclosure, and currency. It is a relatively simplistic model, with a score ranging from 0 to 4 (greater scores are representative of greater quality). 

The HONcode, developed in 1997, is a recognized ethical code for medical websites. This certification is based on eight principles (authority, complementarity, confidentiality, attribution, justifiability, transparency of authorship, honesty in advertising, and editorial policy), and it is freely available to view online. HONcode is a useful tool to remove the burden of evaluating individual sites from the consumer, and it is a mark of accreditation attributed to the websites that meet this code of ethics based on transparent medical information [22].

Statistical analysis

Statistical analysis of the six readability scales was performed for descriptive statistics of the variables of interest, using IBM SPSS Statistics for Windows, Version 25.0 (released 2017, IBM Corp., Armonk, NY). Readability scores, HONcode, and JAMA benchmark criteria are dependent variables. Continuous values are indicated as mean ± SD. The Kruskal-Wallis or the Mann-Whitney U tests were used to compare groups with continuous values. The level of significance was set at 5%, and the confidence intervals (CIs) for parametric distribution were set at 95%.

## Results

A total of 67 patient-oriented educational websites concerning PCL reconstruction were examined for their quality using JAMA benchmark criteria and checking for HONcode seal and analyzed for their readability with six different formulas.

The mean FRE score of the examined websites was 49.33 (SD 11.2) This mean value is significantly lower than the ideal cut-off score of 80, corresponding to a 6th-grade level and reflecting an easy-to-read conversational text. The mean FKG was 8.09 (SD 1.4), and only 55% of the examined websites reflected an easy-to-read text. The mean SMOG score was 7.3 (SD 0.89), the mean CLI was 14.7 (SD 2.2), and the mean ARI was 6.5 (SD 1.4). The results of the three are comparable and confirm that for the right comprehension of the information concerning PCL reconstruction, a high level of education is required. Based on the scores achieved in the different readability formulas, less than 5% of the examined websites can be considered really accessible to the common reader. There was no correlation between Google positioning and readability levels.

The HONcode seal was recorded in only 7.4% of websites; this did not reflect better readability scores; this result is in line with the other educational websites.

The mean JAMA benchmark criteria score was 1.3 (SD 0.7) with no websites scoring 4/4 and 8.9% of websites scoring 0/4. No significant variations in the readability scores were documented in the latter group. The HONcode seal was recorded only in five of the 67 examined websites (7.4%): none of them reported a 4/4 JAMA score; the presence of the HONcode seal was not paradigmatic of a higher JAMA score.

The overall data of each readability score examining the quality of the selected websites are available in Table 3 and Table 4.

**Table 3:** Top PCL educational websites – readability scores (Flesh-Kincaid Reading Ease, Flesh-Kincaid Grade Level, Gunning Fog Score, Simple Measure of Gobbledygook (SMOG) Index, Coleman Liau Index, Automated Readability Index)

Website link	Flesch-Kincaid Reading Ease	Flesch-Kincaid Grade Level	Gunning Fog Score	SMOG Index	Coleman-Liau Index	Automated Readability Index
https://drrobertlaprademd.com/pcl-reconstruction/	54.1	8.4	10.4	8.5	13.2	7.3
https://www.orthovirginia.com/posterior-cruciate-ligament-pcl-reconstruction/	48.6	8.2	11	7.5	15.4	6.9
https://www.ypo.education/orthopaedics/knee/posterior-cruciate-ligament-reconstruction-t59/video/	49.2	8.7	10.7	8.2	13.8	6.9
https://www.physio-pedia.com/PCL_Reconstruction	56.4	7.1	9.1	6.8	13.4	5.4
https://www.wheelessonline.com/joints/posterior-cruciate-ligament-reconstruction/	35.6	11.3	10.3	10.5	13.7	8.2
https://www.mercy.com/health-care-services/orthopedics-sports-medicine-spine/specialties/knee-leg/treatments/pcl-surgery	58.4	6.9	7.9	7.2	12.9	5.3
https://www.orthopaedicsurgery.uci.edu/pcl-reconstruction-orthopaedic-irvine-newportbeach-california.html	57.4	6.4	7	5.7	13.5	4.3
https://manhattansportsdoc.com/pcl-reconstruction-posterior-cruciate-ligament-orthopedic-knee-surgeon-manhattan-new-york-city-ny/	31.5	10	7.5	6.9	19	8.7
https://www.faoconline.com/home/procedures/pcl-reconstruction	49.5	7.6	9.2	6.6	14.9	5.6
https://www.sportssurgerychicago.com/knee/pcl-reconstruction-surgery-westchester-oakbrook-hinsdale-il/	30.1	10.2	7.4	7.1	19.6	9.2
https://noyeskneeinstitute.com/specialties-and-programs/pcl-reconstruction/	47.1	9	10.8	8.6	15.1	8
https://rileywilliamsmd.com/posterior-cruciate-ligament-pcl-reconstruction-repair-manhattan-new-york-city-ny/	53.2	7.8	9.6	7.7	13.7	6.1
https://www.brianforsythemd.com/pcl-reconstruction%E2%80%93allograft.html	53.6	7.8	10.1	7.7	14.3	6.8
https://www.tigerortho.com/pcl-reconstruction-orthopaedic-shoulder-knee-sports-medicine-surgeon-boston-ma.html	47.9	7.9	7.8	6.9	15.8	6.6
https://www.atlanticortho.com/pcl-reconstruction/	53.7	7.8	9.4	7.9	13.3	5.9
https://www.iskinstitute.com/kc/knee/pcl_tear/t3.html	61.5	8.6	11.9	9.2	11.3	8.2
https://srosm.com/orthopedic-specialties/knee/knee-treatments/pcl-reconstruction/	53.6	7.7	9.7	7.6	14.7	6.9
https://www.joecoopermd.com/contents/additional-services/knee/pcl-tear-reconstruction	49.8	7.9	8.8	7	15.8	7
https://www.orthozane.com/Specialties/Knee/Arthroscopic-PCL-Reconstruction/	53.2	7.5	9.8	7.2	14.4	6.1
https://www.wkhs.com/health-resources/wk-health-library/medical-procedures-tests-care-and-management/a-z/posterior-cruciate-ligament-(pcl)-reconstruction	55.8	7.3	9	7.2	13.8	5.9
https://lasportsorthomd.com/pcl-reconstruction-surgery-van-nuys-thousand-oaks-los-angeles-ca/	40.3	8.9	8	7.1	16.7	7.1
https://www.orthobullets.com/knee-and-sports/3009/pcl-injury	48.4	7.6	8.6	6.1	13.6	4.3
https://www.alfredmansourmd.com/posterior-cruciate-ligament-reconstruction-alfred-a-mansour-md-hip-preservation-sports-medicine-surgeon.html	51.4	7.8	8.8	7.3	14.2	6.1
https://www.drlingarajkrishna.com/pcl-reconstruction	46.2	9.1	12.6	8.7	15	7.7
https://matthewprovenchermd.com/pcl-reconstruction-surgery-vail-aspen-denver-co/	44.4	8.4	7.4	6.8	16.8	7.3
https://www.timothybertmd.com/pcl-reconstruction-sports-medicine-scottsdale-phoenix-mesa.html	54.7	7.3	8.7	7	14.1	5.9
https://rothmanortho.com/specialties/treatments/posterior-cruciate-ligament-pcl-reconstruction	41	8.5	7.1	6.2	17.6	7.2
https://orthosportandspine.com/treatments/pcl-reconstruction/	42.9	8.6	7.8	6.9	16.8	7.3
https://www.advancedosm.com/pcl-reconstruction-orthopaedic-sports-medicine-specialist-cypress-houston-tx/	54.3	7.5	8.7	7.2	14.1	6.1
https://www.physio-pedia.com/Posterior_Cruciate_Ligament_Injury	37.9	9.5	11.2	7.6	14.8	6.3
https://www.sports-health.com/sports-injuries/knee-injuries/posterior-cruciate-ligament-pcl-tear-treatment-options	51.1	7.5	8.8	6.9	14.7	5.9
https://www.scottfaucettmd.com/conditions-and-treatments/knee/posterior-cruciate-ligament-reconstruction-washington-dc/	57.9	7.1	8.9	7.3	13	5.4
https://my.clevelandclinic.org/health/diseases/21793-pcl-posterior-cruciate-ligament-tears	47.6	8.2	10.2	7.6	15	6.5
https://www.uptodate.com/contents/posterior-cruciate-ligament-injury	70.8	5.9	8	7.6	11.7	5.8
https://www.choosept.com/guide/physical-therapy-guide-posterior-cruciate-ligament-injury	57	7.5	10.6	8.2	13.6	6.6
https://www.fxrxinc.com/contents/additional-services/knee-treatment/posterior-cruciate-ligament-reconstruction	41.7	9.6	12	8.5	16.5	8.7
https://www.bonsecours.com/health-care-services/orthopedics-sports-medicine/knee/treatments/pcl-surgery	56.7	7.3	9.9	7.6	12.9	5.4
https://www.winchesterhospital.org/health-library/article?id=916896	59.9	6.6	7	6.7	13.7	5.6
https://www.missoulaboneandjoint.com/arthroscopic-posterior-cruciate-ligament-repair-surgery/	59.9	6.4	8.5	6.7	12.3	4.1
https://www.stgeorgesurgical.com/procedure/posterior-cruciate-ligament-pcl-repair/	51.6	7.8	9.9	7.4	14	6
https://medlineplus.gov/ency/patientinstructions/000680.htm	64.7	5.9	8	6.6	11.6	3.8
https://www.shorelineortho.com/specialties/knee-pcl_posterior_cruciate_ligament_injuries_and_reconstruction.php	33.6	9.7	6.6	6.8	19	8.7
https://www.orthopedicsurgeonnyc.com/pcl-reconstruction-surgery	40.4	9.3	9.5	7.8	17	8.2
https://emedicine.medscape.com/article/1252128-overview	46.1	8.4	10.3	7.6	13	4.9
https://centralcoastortho.com/patient-education/posterior-cruciate-ligament-pcl-reconstruction/	24.5	11	6.3	7.2	19.9	9.4
https://www.eliteorthopaedic.com/contents/patient-education/posterior-cruciate-ligament-pcl-tear	63.2	6.1	8.4	6.6	12.2	4.4
https://www.jorgechahlamd.com/knee/pcl-tear-chicago-il/	42.6	9	10.7	8	16	7.4
https://watsonorthopaedics.com/home/health-professional/knee/posterior-cruciate-injury-and-recon/	39.5	9.4	8.7	7.8	16.7	8
https://www.lahey.org/health-library/posterior-cruciate-ligament-pcl-repair/	1.9	14	6.6	8.3	20.9	10
https://www.shouldersandknees.com/posterior-cruciate-ligament-pcl-reconstruction/	58.1	7.3	10.1	7.6	12.8	5.9
https://www.sportssurgerynewyork.com/posterior-cruciate-ligament-pcl-reconstruction-sports-medicine-specialist-ny.html	55.8	7.3	9	7.2	13.8	5.9
https://www.sportsmd.com/sports-injuries/knee-injuries/pcl-tear/	57.4	7.1	9.1	7.2	13.6	5.9
https://www.lmh.org/get-care/orthokansas/knee-leg/pcl-tear-reconstruction/	66.1	6.1	8.7	7	11.6	4.7
https://www.webmd.com/fitness-exercise/posterior-cruciate-ligament-injury	64.2	6	6.4	6.6	12.9	5
https://www.proortho.com/knee/pcl-surgery/	37.5	9	6.3	6.1	17.9	7.5
https://www.marshfieldclinic.org/specialties/orthopedics/knee-surgery/pcl	36.7	10.1	9.1	8.6	18	9.6
https://www.cincysportssurgeon.com/pcl-tears-sports-medicine-orthopedic-surgeon.html	50.3	8.2	9.6	7.9	14.7	6.9
https://robertboykinmd.com/pcl-reconstruction-asheville-north-carolina/	36.3	9.5	8.5	7.1	17.8	8.1
https://www.joionline.net/trending/content/what-recovery-time-pcl-tear	52.1	8.2	9.1	8.1	14.7	7.4
https://www.uofmhealth.org/conditions-treatments/cmc/knee/pcl	54.2	6.9	7.9	6.2	13.6	4.5
https://www.bethesdaccsc.com/pcl-injuries-surgery-center-maryland.html	49.4	8.1	8.9	7.4	14.8	6.6
https://treasurevalleyhospital.com/PCL-Surgery	50.1	7.6	8.1	6.6	14.7	5.7
https://www.ortho-sa.com/pcl-reconstruction-orthopedic-surgeons-san-antonio-tx.html	57.2	6.6	7.8	6.2	13.7	4.9
https://www.angelinaveramd.com/pcl-reconstruction-orthopedic-sports-medicine-specialist-las-vegas-summerlin-henderson.html	52.2	7.9	9.3	7.6	14.1	6.4
https://www.rachelfrankmd.com/pcl-injuries-orthopaedic-surgeon-sports-medicine-specialist-denver-co.html	31.8	9.3	10.2	4.4	18.8	7.3
https://www.howardluksmd.com/pcl-injury-and-surgery-reconstruction/	65.3	6.6	9.2	7.5	10.4	4.6
https://www.katyorthopaedics.com/pcl-reconstruction-dr-volkan-surgeon-fort-bend-cypress-tx.html	58.1	6.4	7.6	6	13.9	4.9

**Table 4:** Top PCL educational websites – JAMA benchmark and HONcode certification

Website link	JAMA authorship	JAMA attribution	JAMA disclosure	JAMA currency	JAMA total	HONcode
https://drrobertlaprademd.com/pcl-reconstruction/	1	0	1	0	2	no
https://www.orthovirginia.com/posterior-cruciate-ligament-pcl-reconstruction/	0	0	0	0	0	no
https://www.ypo.education/orthopaedics/knee/posterior-cruciate-ligament-reconstruction-t59/video/	0	0	0	0	0	no
https://www.physio-pedia.com/PCL_Reconstruction	0	1	1	0	2	no
https://www.wheelessonline.com/joints/posterior-cruciate-ligament-reconstruction/	1	1	1	0	3	no
https://www.mercy.com/health-care-services/orthopedics-sports-medicine-spine/specialties/knee-leg/treatments/pcl-surgery	0	0	1	0	1	no
https://www.orthopaedicsurgery.uci.edu/pcl-reconstruction-orthopaedic-irvine-newportbeach-california.html	0	0	1	0	1	no
https://manhattansportsdoc.com/pcl-reconstruction-posterior-cruciate-ligament-orthopedic-knee-surgeon-manhattan-new-york-city-ny/	1	0	1	0	2	no
https://www.faoconline.com/home/procedures/pcl-reconstruction	0	0	1	0	1	no
https://www.sportssurgerychicago.com/knee/pcl-reconstruction-surgery-westchester-oakbrook-hinsdale-il/	1	0	1	0	2	no
https://noyeskneeinstitute.com/specialties-and-programs/pcl-reconstruction/	1	0	1	0	2	no
https://rileywilliamsmd.com/posterior-cruciate-ligament-pcl-reconstruction-repair-manhattan-new-york-city-ny/	0	0	1	0	1	no
https://www.brianforsythemd.com/pcl-reconstruction%E2%80%93allograft.html	0	0	1	0	1	no
https://www.tigerortho.com/pcl-reconstruction-orthopaedic-shoulder-knee-sports-medicine-surgeon-boston-ma.html	0	0	1	0	1	no
https://www.atlanticortho.com/pcl-reconstruction/	0	0	1	0	1	no
https://www.iskinstitute.com/kc/knee/pcl_tear/t3.html	0	0	0	0	0	no
https://srosm.com/orthopedic-specialties/knee/knee-treatments/pcl-reconstruction/	0	0	0	0	0	no
https://www.joecoopermd.com/contents/additional-services/knee/pcl-tear-reconstruction	0	0	1	0	1	no
https://www.orthozane.com/Specialties/Knee/Arthroscopic-PCL-Reconstruction/	0	0	1	0	1	no
https://www.wkhs.com/health-resources/wk-health-library/medical-procedures-tests-care-and-management/a-z/posterior-cruciate-ligament-(pcl)-reconstruction	0	0	1	0	1	no
https://lasportsorthomd.com/pcl-reconstruction-surgery-van-nuys-thousand-oaks-los-angeles-ca/	0	0	1	0	1	no
https://www.orthobullets.com/knee-and-sports/3009/pcl-injury	0	0	0	0	0	no
https://www.alfredmansourmd.com/posterior-cruciate-ligament-reconstruction-alfred-a-mansour-md-hip-preservation-sports-medicine-surgeon.html	0	0	1	0	1	no
https://www.drlingarajkrishna.com/pcl-reconstruction	0	0	1	0	1	no
https://matthewprovenchermd.com/pcl-reconstruction-surgery-vail-aspen-denver-co/	0	0	1	0	1	no
https://www.timothybertmd.com/pcl-reconstruction-sports-medicine-scottsdale-phoenix-mesa.html	0	0	1	0	1	no
https://rothmanortho.com/specialties/treatments/posterior-cruciate-ligament-pcl-reconstruction	0	0	0	0	0	no
https://orthosportandspine.com/treatments/pcl-reconstruction/	0	0	1	0	1	no
https://www.advancedosm.com/pcl-reconstruction-orthopaedic-sports-medicine-specialist-cypress-houston-tx/	0	0	1	0	1	no
https://www.physio-pedia.com/Posterior_Cruciate_Ligament_Injury	0	1	1	0	2	no
https://www.sports-health.com/sports-injuries/knee-injuries/posterior-cruciate-ligament-pcl-tear-treatment-options	1	0	0	1	2	no
https://www.scottfaucettmd.com/conditions-and-treatments/knee/posterior-cruciate-ligament-reconstruction-washington-dc/	0	0	1	0	1	no
https://my.clevelandclinic.org/health/diseases/21793-pcl-posterior-cruciate-ligament-tears	0	1	1	1	3	Yes
https://www.uptodate.com/contents/posterior-cruciate-ligament-injury	0	1	0	1	2	Yes
https://www.choosept.com/guide/physical-therapy-guide-posterior-cruciate-ligament-injury	1	1	0	1	3	No
https://www.fxrxinc.com/contents/additional-services/knee-treatment/posterior-cruciate-ligament-reconstruction	0	1	1	0	2	No
https://www.bonsecours.com/health-care-services/orthopedics-sports-medicine/knee/treatments/pcl-surgery	0	0	1	0	1	No
https://www.winchesterhospital.org/health-library/article?id=916896	0	1	1	0	2	No
https://www.missoulaboneandjoint.com/arthroscopic-posterior-cruciate-ligament-repair-surgery/	1	0	1	1	3	No
https://www.stgeorgesurgical.com/procedure/posterior-cruciate-ligament-pcl-repair/	0	0	1	0	1	No
https://medlineplus.gov/ency/patientinstructions/000680.htm	1	1	0	1	3	Yes
https://www.shorelineortho.com/specialties/knee-pcl_posterior_cruciate_ligament_injuries_and_reconstruction.php	0	0	1	0	1	No
https://www.orthopedicsurgeonnyc.com/pcl-reconstruction-surgery	0	0	1	0	1	No
https://emedicine.medscape.com/article/1252128-overview	1	0	0	1	2	No
https://centralcoastortho.com/patient-education/posterior-cruciate-ligament-pcl-reconstruction/	0	0	1	0	1	No
https://www.eliteorthopaedic.com/contents/patient-education/posterior-cruciate-ligament-pcl-tear	0	0	1	0	1	No
https://www.jorgechahlamd.com/knee/pcl-tear-chicago-il/	0	0	1	0	1	No
https://watsonorthopaedics.com/home/health-professional/knee/posterior-cruciate-injury-and-recon/	0	0	1	0	1	No
https://www.lahey.org/health-library/posterior-cruciate-ligament-pcl-repair/	0	1	1	1	3	No
https://www.shouldersandknees.com/posterior-cruciate-ligament-pcl-reconstruction/	0	0	1	0	1	No
https://www.sportssurgerynewyork.com/posterior-cruciate-ligament-pcl-reconstruction-sports-medicine-specialist-ny.html	0	0	1	0	1	No
https://www.sportsmd.com/sports-injuries/knee-injuries/pcl-tear/	1	0	1	0	2	No
https://www.lmh.org/get-care/orthokansas/knee-leg/pcl-tear-reconstruction/	0	0	1	0	1	No
https://www.webmd.com/fitness-exercise/posterior-cruciate-ligament-injury	1	1	0	1	3	Yes
https://www.proortho.com/knee/pcl-surgery/	0	0	1	0	1	No
https://www.marshfieldclinic.org/specialties/orthopedics/knee-surgery/pcl	0	0	1	0	1	Yes
https://www.cincysportssurgeon.com/pcl-tears-sports-medicine-orthopedic-surgeon.html	0	0	1	0	1	No
https://robertboykinmd.com/pcl-reconstruction-asheville-north-carolina/	0	0	1	0	1	No
https://www.joionline.net/trending/content/what-recovery-time-pcl-tear	0	0	1	0	1	No
https://www.uofmhealth.org/conditions-treatments/cmc/knee/pcl	0	0	1	0	1	No
https://www.bethesdaccsc.com/pcl-injuries-surgery-center-maryland.html	0	0	1	0	1	No
https://treasurevalleyhospital.com/PCL-Surgery	0	0	0	1	1	No
https://www.ortho-sa.com/pcl-reconstruction-orthopedic-surgeons-san-antonio-tx.html	0	0	1	0	1	No
https://www.angelinaveramd.com/pcl-reconstruction-orthopedic-sports-medicine-specialist-las-vegas-summerlin-henderson.html	0	0	1	0	1	No
https://www.rachelfrankmd.com/pcl-injuries-orthopaedic-surgeon-sports-medicine-specialist-denver-co.html	0	0	1	0	1	No
https://www.howardluksmd.com/pcl-injury-and-surgery-reconstruction/	1	0	1	0	2	No
https://www.katyorthopaedics.com/pcl-reconstruction-dr-volkan-surgeon-fort-bend-cypress-tx.html	0	0	1	0	1	No

## Discussion

In the last decades, we have experienced a logarithmic growth of information-seeking individuals looking for a solution to manage the most different pathologies [23]. A significant growth of online educational materials has been appreciated in parallel, with a beneficial effect on health professionals and patients. Anyway, this rise in “online information production” is not automatically indicative of a rise in quality and accessibility for the average user. Scientific dissemination needs to translate technical details into a simple and comprehensive language. Furthermore, clear information needs to be supported by a solid scientific basis and authority of the sources, which should be publicly expressed. The Internet has become the first meeting place between patients and health professionals: the care for details should not be limited to health services (in the strict sense) but should be reserved for the educational context, too.

On this premise, the readability and quality of online patient educational material concerning PCL reconstruction were investigated. The findings of the present study were in concordance with prior studies focusing on orthopedic educational tools [9,11,24-29] and show that the most frequently accessed patient educational sources generally exceed the reading ability of the average US reader and the readability recommendations of the American Medical Association (AMA) and National Institutes of Health (NIH) [30]. The most frequently accessed online patient educational material regarding PCL reconstruction is not always easily accessible to the average reader, requiring much more than a 6th-grade reading level (the cut-off educational value recommended by the AMA and NIH for a comprehensible document) [31-35]. Only 4.4% of the examined websites met this condition, while 50.8% required an 8th-grade reading level and 44.8% required a set of graduate skills. This feedback stresses a mismatch between the recommended versus the practical reading levels of online educational tools related to PCL reconstruction, limiting the value of the information and full participation in the decision-making process.

Online patient educational tools aim to get adequate comprehension and informed decisions, thus supporting both health professionals and patients. The limited office-visit time frames and the need to process medical information promote this new way of communication on the Internet, which is always available and easily accessible. Anyway, it is essential not only to get but also to comprehend the information received. This is unavoidably important in healthcare, especially in surgery, considering the complex treatment modalities involved and the medicolegal implications. Online instruments should adequately support the in-presence relationship between the patient and the orthopedic surgeon. On this basis, we have also examined the quality of the information with two instruments, the HONcode and JAMA benchmark criteria. The HONcode certification is the most used ethical code for health-related information available on the Internet, designed for patients, health professionals, and webmasters. It is an ethical standard assigned to websites following eight procedural principles and publishing transparent information in order to have useful, objective, and correct data.

A significant limit is determined by the voluntary request submitted by the website manager to get HONcode certification [36]. The JAMA benchmark criteria aim to pursue a similar goal, by analyzing the authoritativeness of a website. It is a simple tool that can be easily used by a common reader to encounter the strength of the sources based on authorship, attribution, disclosure, and currency. The present study showed a weak correlation between the HONcode certification and the JAMA benchmark score: the presence of the HONcode seal was not paradigmatic of a higher JAMA score.

Moreover, a critical evaluation to compare the readability and quality of the examined websites was performed by analyzing the results of the different scores and crossing the data to achieve as truthful as possible conclusions. Despite this statement of intent, the objective analysis of the online patient educational material concerning PCL reconstruction did not achieve the objectives set, requiring a high level of education to grant adequate comprehension. The average reader rarely explores websites indexed from the third Google® page onwards, and the top 5 Google® results get almost 70% of all clicks [37]. Anyway, Google® rank is not paradigmatic of the quality of content and readability/understandability of the information provided. Search algorithms look at many factors, including not only the quality of content but also the page experience (on-page optimization, mobile-first optimization, and page speed) and the presence of internal and external links. All these aspects can be misleading especially when someone needs to address an issue as complex as health management.

The employment of six scales for readability and two tools for quality analysis strengthens the methodological architecture of the study, minimizing any potential bias between scales. The results are consistent with other previous research examining orthopedic patient education materials, thus confirming the poor accessibility of these sources to the average readers [7,8,38-41]. This is even more inconvenient since a poor understanding is a premise for poor therapeutic adherence and worse clinical results, limiting patients' and health professionals' satisfaction [31,42,43]. On these premises, the decision-making process is unavoidably affected by the absence of a detailed discussion regarding complications and risks. This is very important when discussing and explaining the surgical procedure since risks and complications are the key aspects of informed consent as explicated by the American College of Surgeons and the American Academy of Orthopaedic Surgeons (AAOS) several times [44-49].

Although the aforementioned findings, the present study has some limits. First, although we are aware that PCL reconstruction is necessary only for a small proportion of patients, we decided to focus only on surgical management. A surgical procedure generally requires more consciousness and participation by patients (who are more prone to deepen the theme), and we were curious to find out whether online educational websites concerning PCL surgical reconstruction would ensure clear and accurate information. A subsequent evaluation of the educational websites concerning conservative management may be considered in a further study. Second, the search analyzed only websites written in English and not peer-reviewed. Search engine trends suffer from temporal and geographical variations, so subtle variations can be encountered by changing the query parameters. The anonymous modality used for our search on Google® should limit further confounding factors determined by previous research. Third, the website list might theoretically differ by using other search engines; anyway, given the striking dominant position of Google® in the Internet search query market [50], we believe that our choice does not change the key message of this study. We think that our googling can be easily considered the mirror of the online results of the general population. Fourth, like other similar studies, although we are aware that the use of different readability tools should grant an adequate approximation of the complexity of this topic, we did not get practical feedback from a group of patients accessing the information hosted on these websites. Further studies might compare readability formulae scores and practical patient-understandability feedback.

## Conclusions

The present study emphasized the limits of online patient educational materials concerning PCL reconstruction. The readability of the examined websites was too complex and should therefore adapted to favor adequate comprehension by the average reader and to satisfy the aim for which they are intended. Good-quality information is another important objective to achieve, and unfortunately, this study highlighted the poor methodological quality of the available resources on PCL reconstruction. With a topic of great interest and an increasing number of patients searching for their health information on the Internet, the improvement in the quality of communication and optimization in readability would be necessary to grant adequate comprehension to an ever-growing audience.

Practice implications

Online medical information has been shown to influence patient healthcare decision processes; for this reason, patient-oriented educational materials should be clear and easy to understand. This is the first study that assessed the readability and quality of online patient educational materials regarding posterior cruciate ligament reconstruction. The results of this study should promote a willingness to make patient educational website content on this focus easily accessible to the average reader.

## References

[1] O'Connor AM, Légaré F, Stacey D (2003). Risk communication in practice: the contribution of decision aids. BMJ.

[2] Kıvrak A, Ulusoy İ (2023). How high is the quality of the videos about children's elbow fractures on Youtube?. J Orthop Surg Res.

[3] Ziebland S, Wyke S (2012). Health and illness in a connected world: how might sharing experiences on the internet affect people's health?. Milbank Q.

[4] Townsend A, Leese J, Adam P, McDonald M, Li LC, Kerr S, Backman CL (2015). eHealth, participatory medicine, and ethical care: a focus group study of patients’ and health care providers’ use of health related Internet information. J Med Internet Res.

[5] Ulusoy I, Yılmaz M, Kıvrak A (2023). How efficient is ChatGPT in accessing accurate and quality health-related information?. Cureus.

[6] (2024). Eligibility.com. The most googled medical symptoms by state. https://eligibility.com/medicare/states-most-googled-medical-symptom.

[7] Roberts H, Zhang D, Dyer GS (2016). The readability of AAOS patient education materials: evaluating the progress since 2008. J Bone Joint Surg Am.

[8] Eltorai AE, P Thomas N, Yang H, Daniels AH, Born CT (2016). Readability of trauma-related patient education materials from the American Academy of Orthopaedic Surgeons. Trauma Mon.

[9] Abdullah Y, Alokozai A, O'Connell S, Mulcahey MK (2022). Online patient education materials for common sports injuries are written at too-high of a reading level: a systematic review. Arthrosc Sports Med Rehabil.

[10] Chapman L, Brooks C, Lawson J, Russell C, Adams J (2019). Accessibility of online self-management support websites for people with osteoarthritis: A text content analysis. Chronic Illn.

[11] Broderick JM, McCarthy A, Hogan N (2021). Osteotomy around the knee: assessment of quality, content and readability of online information. Knee.

[12] Venosa M, Tarantino A, Schettini I, Padua R, Cifone MG, Calvisi V, Romanini E (2021). Stem cells in orthopedic web information: an assessment with the DISCERN Tool. Cartilage.

[13] Karunaratne S, Harris IA, Trevena L, Horsley M, Fajardo M, Solomon M (2021). Online decision aids for knee arthroplasty: an environmental scan. JBJS Rev.

[14] Goff AJ, Barton CJ, Merolli M, Zhang Quah AS, Ki-Cheong Hoe C, De Oliveira Silva D (2023). Comprehensiveness, accuracy, quality, credibility and readability of online information about knee osteoarthritis. Health Inf Manag.

[15] Griffiths SZ, Albana MF, Bianco LD, Pontes MC, Wu ES (2021). Robotic-assisted total knee arthroplasty: an assessment of content, quality, and readability of available internet resources. J Arthroplasty.

[16] Murphy B, Irwin S, Condon F, Kennedy C (2022). Readability and quality of online information for patients pertaining to revision knee arthroplasty: an objective analysis. Surgeon.

[17] Geiger G, Aliyev RM (2013). Treatment costs for anterior cruciate ligament reconstruction: procedure related cost analysis in an university hospital [Article in German]. Unfallchirurg.

[18] Owesen C, Aas E, Årøen A (2018). Surgical reconstruction is a cost-efficient treatment option for isolated PCL injuries. Knee Surg Sports Traumatol Arthrosc.

[19] O'Neill SC, Baker JF, Fitzgerald C, Fleming C, Rowan F, Byrne D, Synnott K (2014). Cauda equina syndrome: assessing the readability and quality of patient information on the Internet. Spine (Phila Pa 1976).

[20] (2024). Readability test - WebFX. https://www.webfx.com/tools/read-able/.

[21] Silberg WM, Lundberg GD, Musacchio RA (1997). Assessing, controlling, and assuring the quality of medical information on the Internet: caveant lector et viewor--Let the reader and viewer beware. JAMA.

[22] Boyer C, Baujard V, Geissbuhler A (2011). Evolution of health web certification through the HONcode experience. Stud Health Technol Inform.

[23] Fox S, Rainie L (2013). The online health care revolution: how the web helps Americans take better care of themselves. https://www.pewresearch.org/internet/2000/11/26/the-online-health-care-revolution/.

[24] Abdullah Y, Alokozai A, Mathew AJ, Stamm MA, Mulcahey MK (2022). Patient education materials found via Google search for shoulder arthroscopy are written at too-high of a reading level. Arthrosc Sports Med Rehabil.

[25] Akinleye SD, Krochak R, Richardson N, Garofolo G, Culbertson MD, Erez O (2018). Readability of the most commonly accessed arthroscopy-related online patient education materials. Arthroscopy.

[26] Yi PH, Ganta A, Hussein KI, Frank RM, Jawa A (2013). Readability of arthroscopy-related patient education materials from the American Academy of Orthopaedic Surgeons and Arthroscopy Association of North America Web sites. Arthroscopy.

[27] Ghodasra JH, Wang D, Jayakar RG, Jensen AR, Yamaguchi KT, Hegde VV, Jones KJ (2018). The assessment of quality, accuracy, and readability of online educational resources for platelet-rich plasma. Arthroscopy.

[28] Shnaekel AW, Hadden KB, Moore TD, Prince LY, Lowry Barnes C (2018). Readability of patient educational materials for total hip and knee arthroplasty. J Surg Orthop Adv.

[29] Doinn TÓ, Broderick JM, Abdelhalim MM, Quinlan JF (2020). Readability of patient educational materials in hip and knee arthroplasty: has a decade made a difference?. J Arthroplasty.

[30] Eltorai AE, Sharma P, Wang J, Daniels AH (2015). Most American Academy of Orthopaedic Surgeons' online patient education material exceeds average patient reading level. Clin Orthop Relat Res.

[31] Berkman ND, Sheridan SL, Donahue KE, Halpern DJ, Crotty K (2011). Low health literacy and health outcomes: an updated systematic review. Ann Intern Med.

[32] Kim H, Xie B (2017). Health literacy in the eHealth era: a systematic review of the literature. Patient Educ Couns.

[33] Szabó P, Bíró É, Kósa K (2021). Readability and comprehension of printed patient education materials. Front Public Health.

[34] Beaunoyer E, Arsenault M, Lomanowska AM, Guitton MJ (2017). Understanding online health information: evaluation, tools, and strategies. Patient Educ Couns.

[35] Kreps GL (2018). Promoting patient comprehension of relevant health information. Isr J Health Policy Res.

[36] (2024). Health on the Net Foundation. https://en.wikipedia.org/wiki/Health_On_the_Net_Foundation.

[37] Meyer C (2019). The top 5 results in Google get almost 70% of all clicks. https://www.advance-metrics.com/en/blog/the-top-5-results-in-google-get-almost-70-of-all-clicks/.

[38] Gao B, Shamrock AG, Gulbrandsen TR, O'Reilly OC, Duchman KR, Westermann RW, Wolf BR (2022). Can patients read, understand, and act on online resources for anterior cruciate ligament surgery?. Orthop J Sports Med.

[39] Long WW, Modi KD, Haws BE, Khechen B, Massel DH, Mayo BC, Singh K (2018). Assessing online patient education readability for spine surgery procedures. Clin Spine Surg.

[40] Mehta MP, Swindell HW, Westermann RW, Rosneck JT, Lynch TS (2018). Assessing the readability of online information about hip arthroscopy. Arthroscopy.

[41] Zhang D, Schumacher C, Harris MB (2016). The quality and readability of internet information regarding clavicle fractures. J Orthop Sci.

[42] Dewalt DA, Berkman ND, Sheridan S, Lohr KN, Pignone MP (2004). Literacy and health outcomes: a systematic review of the literature. J Gen Intern Med.

[43] Miller TA (2016). Health literacy and adherence to medical treatment in chronic and acute illness: a meta-analysis. Patient Educ Couns.

[44] Long KL, Ingraham AM, Wendt EM, Saucke MC, Balentine C, Orne J, Pitt SC (2021). Informed consent and informed decision-making in high-risk surgery: a quantitative analysis. J Am Coll Surg.

[45] (2024). American College of Surgeons. Statements on principles. https://www.facs.org/about-acs/statements/statements-on-principles/.

[46] Fraval A, Chandrananth J, Chong YM, Coventry LS, Tran P (2015). Internet based patient education improves informed consent for elective orthopaedic surgery: a randomized controlled trial. BMC Musculoskelet Disord.

[47] Robb WJ III, Carroll C IV, Kuo C (2013). Orthopaedic surgical consent: the first step in safety: informed consent is critical for improving surgical safety and quality. AAOS Now.

[48] Shemesh S, Sidon E, Heller S (2019). The quality of informed consent obtained for orthopedic surgeries-elective versus trauma: A prospective interview-based study. J Orthop Surg (Hong Kong).

[49] (2024). American Academy of Orthopaedic Surgeons (AAOS) - Information Statement Orthopaedic Surgical Consent. https://www.aaos.org/globalassets/about/bylaws-library/information-statements/1038-orthopaedic-surgical-consent.pdf.

[50] (2024). Search engine market share in 2023. https://www.oberlo.com/statistics/search-engine-market-share.

